# Management of Ventricular Arrhythmias in Patients with Left Ventricular Assist Devices: Pathophysiology, Risk Stratification, and Ablation Strategies

**DOI:** 10.3390/jcm14186604

**Published:** 2025-09-19

**Authors:** Giuseppe Sgarito, Francesco Campo, Sergio Sciacca, Michele Pilato, Manlio Cipriani, Sergio Conti

**Affiliations:** 1 IRCCS Istituto Mediterraneo per i Trapianti e Terapie ad Alta Specializzazione, University of Pittsburgh Medical Center, 90127 Palermo, Italy; 2 Division of Cardiology, Department of Internal Medicine, The Carver College of Medicine, University of Iowa Health Care Center, University of Iowa, Iowa, IA 52242, USA

**Keywords:** Ventricular Arrhythmias, Left Ventricular Assist Device (LVAD), Catheter Ablation, Electrical Storm, Heart Failure

## Abstract

Ventricular arrhythmias (VAs) are common and clinically important complications in patients supported by left ventricular assist devices (LVADs), occurring in up to 50% of cases within the first year after implantation. Despite the hemodynamic support provided by LVADs, VAs are linked to increased morbidity and mortality, primarily through recurrent implantable cardioverter defibrillator (ICD) shocks and right ventricular failure. The underlying mechanisms of VAs in this population are multifactorial, involving structural myocardial remodeling, device-related factors, and pre-existing arrhythmic substrates. Catheter ablation has become a valuable treatment option when antiarrhythmic drug therapy and device reprogramming are inadequate, though procedural timing (pre-, intra-, or post-implantation) and approaches remain under discussion. Epicardial access during LVAD surgery may provide advantages for selected patients, while ablation after implantation poses technical challenges due to altered anatomy and electromagnetic interference. This review offers a comprehensive overview of the epidemiology, pathophysiology, risk stratification, and management of VAs in LVAD recipients, emphasizing technical considerations, procedural safety, and clinical outcomes of catheter ablation. A multidisciplinary approach remains essential in guiding personalized treatment and optimizing outcomes for this complex population. Undergoing studies will provide more insight into optimal management of arrhythmias, particularly regarding the optimal timing of catheter ablation. The impact of new technologies such as non-invasive mapping alongside pre-procedural imaging needs also to be further evaluated.

## 1. Introduction

Left ventricular assist devices (LVADs) are increasingly used for managing patients with advanced and refractory heart failure (HF), serving as a bridge to cardiac transplantation (BT) or as destination therapy (DT) for those who do not meet transplantation criteria. Although LVADs have been demonstrated to improve survival in this setting of patients, arrhythmias, and in particular ventricular arrhythmias (VAs), are common, with a prevalence ranging between 20 and 50% during the first year after LVAD implantation [1,2]. Some authors consider VAs the second most frequent complication after surgery [3]. Despite the hemodynamic support provided by LVADs, VAs significantly contribute to increased morbidity through recurrent implantable cardioverter-defibrillator (ICD) shocks and higher mortality among LVADs recipients. Although there are numerous reports of sustained VAs—such as ventricular tachycardia (VT) and ventricular fibrillation (VF)—in patients with LVAD, these arrhythmias have detrimental effects on LVAD efficacy, leading to cardiac failure due to right ventricular failure, especially in the presence of pulmonary hypertension [4,5,6,7]. In the Second Interagency Registry for Mechanical Circulatory Support (INTERMACS) study, VAs represent the leading cause of mortality post-implantation [8].

Understanding the epidemiology, mechanisms, and management of VAs is crucial for improving patient outcomes. The pathophysiology of VAs in LVAD patients is multifactorial. Structural changes associated with end-stage HF, such as myocardial ischemia, necrosis, and fibrotic remodeling, coexist with device-related alterations, creating substrates that promote arrhythmogenesis. Previous studies indicate that a history of VAs before LVAD implantation significantly increases the risk of post-operative VAs, emphasizing the importance of comprehensive pre-operative assessments [9]. Notably, a significant difference in mortality has been reported among patients with early post-operative VAs when LVAD was implanted as DT compared to BTT, probably reflecting a more compromised overall clinical condition [10]. Antiarrhythmic drugs (AADs) are first-line therapy both in the acute and chronic settings. Besides beta-blockers, mainly amiodarone and other medications such as sotalol, mexiletine, and intravenous sodium channel blockers like lidocaine and procainamide may be considered on an individual basis [11].

Patients with recurrent VAs and end-stage HF may be considered for LVAD implantation and catheter ablation. The optimal timing for treatment and whether catheter ablation should be performed before or after LVAD implantation remains uncertain. Several factors should be assessed in the decision-making process in the setting of a multidisciplinary team including HF specialists, electrophysiologists, cardiothoracic surgeons, nurses, and a LVAD coordinator [12]. Catheter ablation of VAs was reported more than a decade ago among LVAD recipients. In a recent Consensus Statement, catheter ablation is recommended in cases of recurrent VAs refractory to AADs, after ICD reprogramming, and following the exclusion of removable causes (e.g., mechanical arrhythmias secondary to LVAD cannula irritation) [2]. In this patient population, catheter ablation involves specific technical and procedural variables with uncertain impact on patient outcomes. There is limited quality data on the use of devices, ablations, and the implementation of guideline-directed HF medical therapies in patients with LVADs. The current evidence is limited due to the observational nature of the studies, relatively small sample sizes, and the use of various LVAD models. This review highlights the primary features of VAs in LVAD patients, with an emphasis on device programming, technical aspects of catheter ablation and both procedural and clinical outcomes.

## 2. Search Strategy and Study Selection

A comprehensive literature search was conducted between 2000 and 2025 using the databases PubMed, Scopus, and Google Scholar. The search strategy combined the following keywords: (“ventricular arrhythmias” OR “ventricular tachycardia” OR “ventricular fibrillation” OR “electrical storm”) AND (“left ventricular assist device”) AND (“implantable cardioverter defibrillator” OR “cardiac resynchronization therapy” OR “antiarrhythmic drugs” OR “catheter ablation”) AND (“heart failure” OR “end-stage heart failure”). Authors independently screened titles and abstracts, followed by a full-text assessment. Disagreements were resolved by consensus. A total of 66 studies met the inclusion criteria and were included in the review.

## 3. LVAD—General Structure and Functional Mechanisms

**Figure 1:**
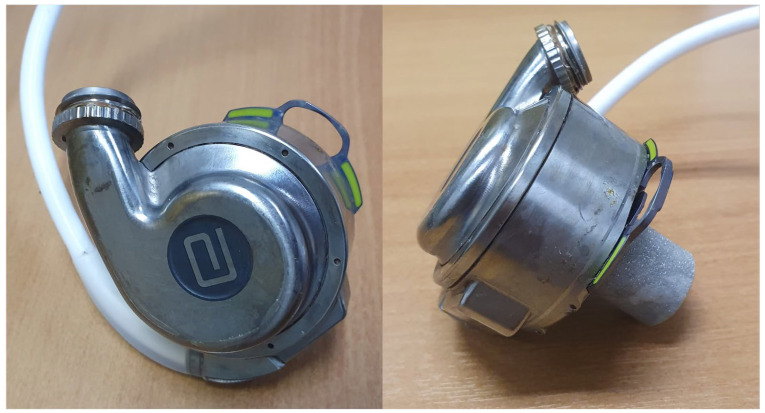
A fully magnetically levitated centrifugal-flow pump.

LVADs are a heterogeneous class of mechanical circulatory support devices increasingly used to treat end-stage HF. Over recent decades, more than 50,000 LVADs have been implanted worldwide. Their primary function is to enhance cardiac output by increasing the ejection of blood from the left ventricle (LV) into the systemic circulation. The most recent generation of LVADs comprises continuous-flow pumps featuring a central electromagnetically driven centrifugal rotor. The electromagnetic levitation rotor reduces friction and wear, thereby enhancing the device’s efficiency and lifespan. Additionally, thanks to magnetic levitation, these devices minimize direct contact between the mechanical parts and the patient’s blood, reducing the risk of thrombosis and improving safety and reliability of the device [13]. The standard functional components of any currently available LVAD include an inflow cannula, a pump, an outflow cannula, a driveline, and a controller (Figure 1).

Blood flow remains steady throughout the cardiac cycle, from the inlet cannula at the LV apex to the outflow cannula connected to the ascending aorta. It is directly proportional to the pump speed and inversely related to the pressure difference between the pump’s inlet (LV pressure) and outlet (aortic pressure) orifices [14].

**Figure 2:**
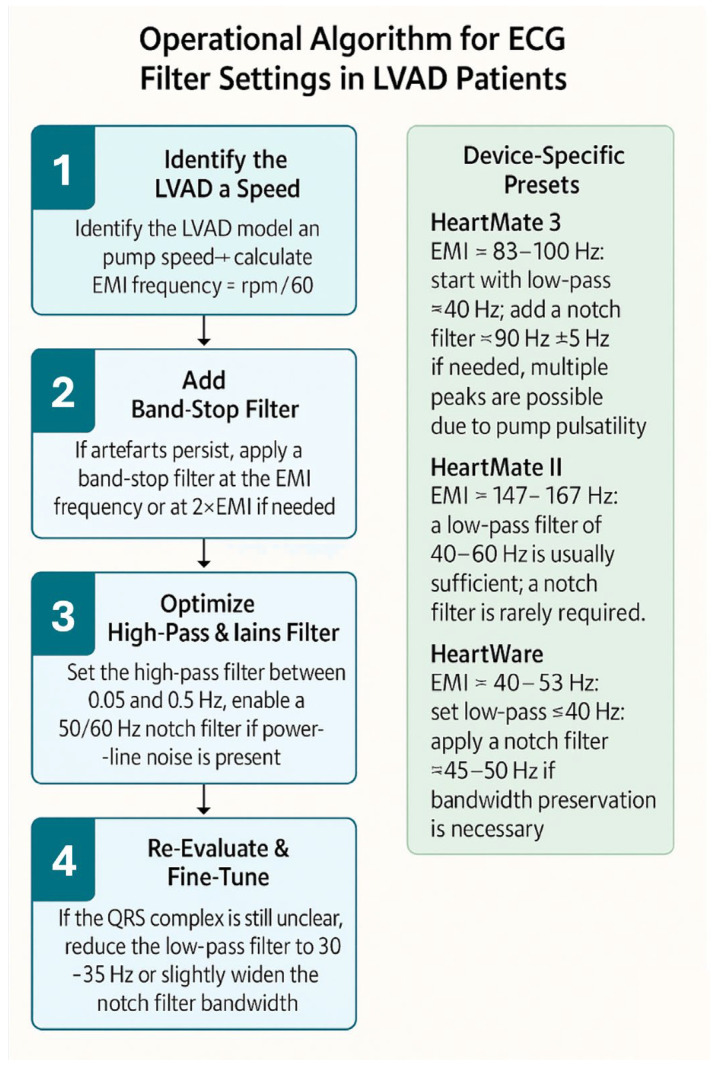
Operational algorithm for ECG filter settings in patients with left ventricular assist device.

All LVADs produce electromagnetic interference (EMI), which appears as high-frequency noise on standard 12-lead ECG recordings. LVADs EMI is primarily driven by the impeller’s rotational speed; however, different LVAD models exhibit varying minor EMI components because of their design differences. The HeartMate 3 by Abbott, for example, operates at impeller rotational speeds ranging from 5000 to 6000 rpm, corresponding to oscillating frequencies of 83.3 to 100 Hz. Adjusting the low-pass filter to 40 Hz can effectively remove the signals causing these artefacts, normalizing the isoelectric baseline, thereby enhancing the readability and overall quality of the ECG and facilitating rhythm identification [15]. This comes with the tradeoff of discarding high-frequency components, which, for example, contribute to the QRS morphology and may carry clinical value. By adding an adjustable band-stop style filter for patients with LVADs, which blocks a band of frequencies around the main oscillation frequency causing EMI, we can more effectively and specifically exclude LVAD-related interference while preserving the high-frequency components of the ECG. Table 1 summarizes the typical pump speeds, corresponding EMI frequencies, and recommended filter settings to improve ECG clarity in various devices. An operational algorithm for ECG filter settings is shown in Figure 2.

**Figure 3:**
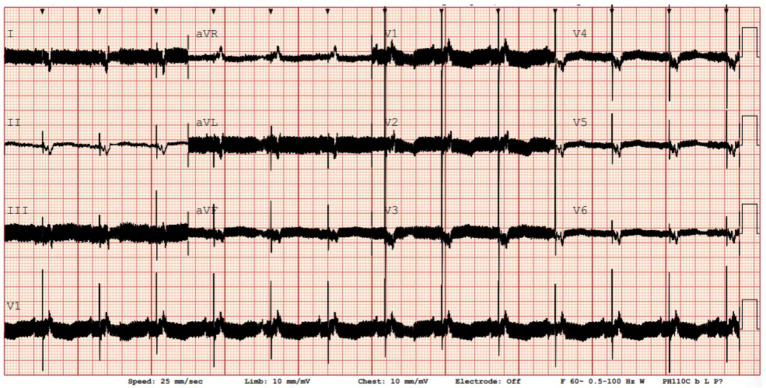
12-lead ECG with standard filters in sinus rhythm showing electromagnetic interference in LVAD recipient.

**Figure 4:**
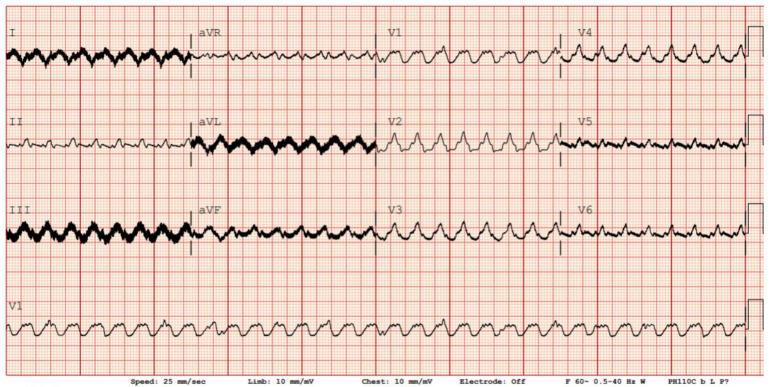
12-lead ECG of a ventricular tachycardia in the same patient with ad hoc filters to reduce the electromagnetic interference produced by the LVAD.

A low-pass filter set around 40 Hz is generally effective; in contrast, device-specific band-stop (“notch”) filters can be applied when artefacts persist, particularly in HeartMate 3, which often produces multiple pulsatility-related frequency peaks, therefore requiring more tailored filtering strategies (Figure 3 and Figure 4) [16].

## 4. Etiology of Ventricular Arrhythmias in Patients with LVAD

VAs occurring mainly in the early post-operative period are commonly linked to hyperactivity of the sympathetic nervous system, fluid depletion or overload, electrolyte imbalances, and the use of inotropic drugs [17]. Additionally, during the first week after LVAD implantation, the immediate hemodynamic effects of LV unloading led to a temporary reduction in QRS duration and an increase in both absolute and heart rate-corrected QT interval, acting as a potential electrical substrate for VAs [18]. However, monomorphic sustained VT is the most frequent arrhythmia, occurring in approximately 85% of cases, followed by ventricular fibrillation (VF), reported in nearly 30% of cases [19,20]. The monomorphic morphology suggests a reentrant mechanism related to preexisting scars or the formation of new scars from the implantation of the inlet cannula directly into the LV apex, which inevitably becomes another potential source of arrhythmias [17]. Nonetheless, Sacher et al. observed that the primary substrate of VAs was mainly related to pre-existing myocardial scar rather than the scar associated with the LVAD inlet cannula itself, with only 9% of targeted VTs linked to the LVAD cannula [21]. Similar findings were reported by Anderson et al., where scar-related reentry was the predominant mechanism for VT (90%), with VAs related to the inlet cannula occurring in 19% of cases [22]. These results were also confirmed by a large study involving over 600 LVAD patients, documenting monomorphic VT in 91% of cases and four cases of polymorphic VT or VF. Electroanatomical mapping of these VAs revealed that a reentrant mechanism related to the native scar was far more common (75%) than that involving the apical inflow cannulation site (14%), focal or micro-reentry VT (7%), or bundle branch reentry (3.5%) [23]. Finally, in a minority of patients, accounting for 3%, the arrhythmia mechanism is due to suctioning—a mechanical contact between the inlet cannula and the LV wall [21]. Several causes, including excessive fluid depletion or VAs themselves, can lead to a reduction in cardiac preload, resulting in suction. This results in a decreased volume of blood available to the pump, leading to reduced blood flow. If there is a significant decrease in the cardiac preload, while maintaining a constant rotational speed, the device induces further volume unloading and can become partially or fully suctioned to the LV walls, arresting forward flow. The suction can mechanically trigger the onset of VAs, further worsening the vicious cycle of volume loss and device dysfunction. Therefore, VAs contribute to LVAD dysfunction, and in certain adverse hemodynamic conditions, the LVAD itself can induce VAs [24].

Several studies have examined risk factors that can predict early and late VAs following LVAD implantation. The strongest predictors of early VAs after LVAD implantation are a pre-implant history of VAs, pre-implant atrial fibrillation, HF lasting over 12 months, and the absence of therapy with ACE inhibitors and beta-blockers [9,25,26,27,28]. Similarly, history of VAs, lack of treatment with ACE inhibitors, HF duration exceeding 12 months, early VAs after implantation, history of atrial fibrillation, and non–ischemic cardiomyopathy were identified in the VT-LVAD score as predictors of late VAs [29].

VAs are also common in the pediatric population, as demonstrated in a recent large study investigating the arrhythmic burden in pediatric patients with LVAD [30]. In particular, patients diagnosed with cardiomyopathy or myocarditis are more likely to develop VAs compared to those with congenital heart disease or those with an arrhythmia before LVAD placement.

## 5. LVAD and Cardiac Implantable Electronic Devices

**Figure 5:**
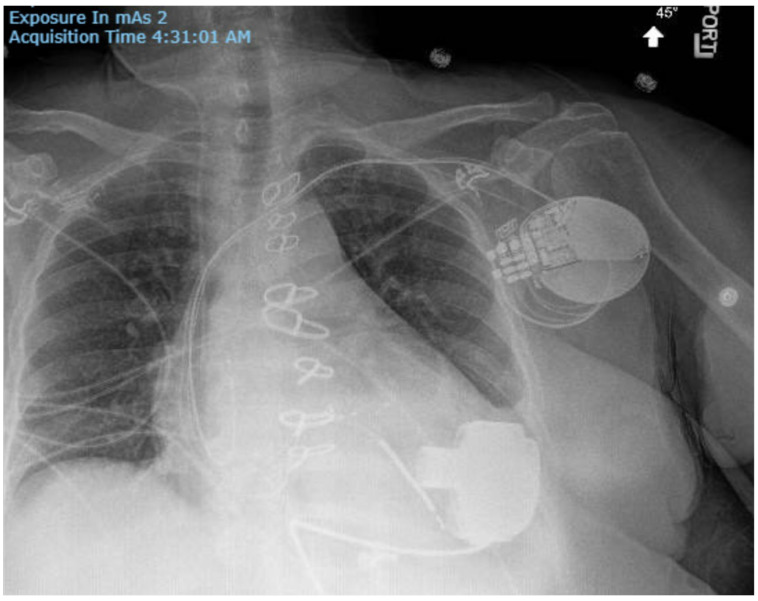
Chest X-ray. Posterior–anterior view. Patient with a transvenous cardiac resynchronization therapy-defibrillator and a left ventricular assist device.

Implantable cardioverter-defibrillators (ICDs) and cardiac resynchronization therapy with defibrillators (CRT-Ds) are the most common cardiac implantable electronic devices (CIEDs) among the LVAD population (Figure 5). ICD implantation is advised for advanced HF patients who may also be candidates for LVAD or heart transplantation; however, its impact on survival remains uncertain. If it is true that patients with prior ICD or CRT-D implantation may undergo LVAD implantation, de novo ICD implantations in patients with previously implanted LVAD are controversial, and their use has gradually declined [31].

The first data on ICD impact in patients with the previous generation of LVADs—pulsatile flow—comes from the PCHF-VAD registry. In this multicenter European registry, patients with an ICD had a 36% reduction in mortality in a multivariate analysis, with a mean follow-up of 1.1 years. The occurrence of VAs in LVAD patients increased the risk of both all-cause and cardiovascular death, while an active ICD device resulted in a reduction of almost 50%. This study supported the conclusion that ICD implantation was associated with significantly better survival in LVAD patients [32]. Surprisingly, in the INTERMACS Registry, the comparison between a group of 2209 ICD patients and a propensity-score-matched group without an ICD did not support the effectiveness and prognostic benefits of ICD in patients with newer-generation continuous-flow LVADs. Indeed, the ICD group had an increased mortality risk, an increased incidence of heart transplantation, and an increased number of hospitalizations due to VAs [33]. Finally, in a meta-analysis examining the additive role of ICD in prolonging survival in HF patients supported with continuous-flow LVAD, implanted as DT or BT, there was no survival benefit of active ICD therapy [34]. These inconsistent data, specifically the lack of documented survival benefit among continuous-flow LVAD recipients, in conjunction with the likelihood of VAs hemodynamic tolerance and the associated risks of ICD implant in these patients, favor an individualized approach. Indeed, the 2022 ESC guidelines for the management of patients with ventricular arrhythmias and the prevention of sudden cardiac death recommend ICD implantation in LVAD patients with symptomatic sustained VAs in class IIa-B [35]. Wearable cardioverter–defibrillators could be an alternative in selected cases of end-stage HF, limited to patients on the urgent waiting list for heart transplant who are not already carriers of an ICD [35].

Finally, optimal device programming is crucial in managing patients with LVADs, both in terms of pacing and VA therapies. Aggressive or standard ICD programming regarding shock discharge can impair quality of life. Frequent ICD shocks are associated with worse long-term outcomes, poorer quality of life, and adverse psychological effects [36]. More conservative programming should be considered for managing ICD in LVAD patients, with longer detection zones and a preference for ATP over shocks.

If it has been already demonstrated how to manage VA therapies in LVAD recipients, there is conflicting evidence regarding the pacing modalities, particularly in previously implanted CRTs. Recent data from a prospective randomized crossover study may support switching off CRT pacing. The study showed that RV pacing was associated with significantly improved functional status and quality of life and reduced VAs [37]. However, other data support the superiority of CRT pacing over other pacing modalities in terms of acute improvement of RV contractility in patients with LVADs [38].

## 6. Antiarrhythmic Drug Therapy in Patients with LVAD and Ventricular Arrhythmias

VAs should still be treated promptly, as they may lead to worsening clinical outcomes and increased mortality, even though the LVAD may provide adequate hemodynamic support during VAs. Due to the uniqueness and size of the LVAD cohort, current recommendations for AAD therapy are based on data from HF patients without LVAD. It remains unclear whether, in LVAD patients, it is feasible and safe to use AADs that are otherwise contraindicated in HF patients without LVAD [2].

In the acute setting, the preferred AADs are intravenous beta-blockers, amiodarone, and sodium channel blockers such as lidocaine and procainamide [39]. For long-term management, oral beta-blockers and amiodarone are commonly used, while additional pharmacological options for preventing recurrent VAs include mexiletine and dofetilide [40]. However, although AADs may relieve symptoms and potentially decrease the number of ICD interventions, none of the currently available medications have shown a significant impact on mortality reduction [41]. In patients on amiodarone treatment before LVAD implant, a higher mortality rate was observed compared to those in whom amiodarone treatment was initiated after the LVAD implant (32.9% vs. 29.6%; *p* = 0.008) [42]. It remains unclear whether amiodarone started before LVAD implantation should be discontinued afterward, as available data is limited. In one study, discontinuation of amiodarone after LVAD implantation led to increased recurrence of arrhythmias but not increased mortality [43]. Early short-term prophylactic use of amiodarone after LVAD implantation may be beneficial but there is no evidence to support this strategy. Initiation of amiodarone after LVAD implantation was not associated with a lower incidence of VAs but did result in a reduction in atrial arrhythmias during follow-up [44].

Finally, it is still unclear whether other medications commonly prescribed for HF patients, which have been shown to reduce the burden of arrhythmias, will have similar effects in patients with a LVAD. Sacubitril-Valsartan, an angiotensin receptor-neprilysin inhibitor (ARNI), has demonstrated a reduction in the combined endpoint of death and HF hospitalization in patients with HF with reduced ejection fraction compared to enalapril [45]. Some small studies have suggested that ARNI may possess antiarrhythmic properties. However, the mechanism by which ARNI may reduce the incidence of VAs is still debated; it could be due to structural reverse remodeling or a direct impact on ventricular conduction properties [46].

## 7. Catheter Ablation of Ventricular Arrhythmias

**Figure 6:**
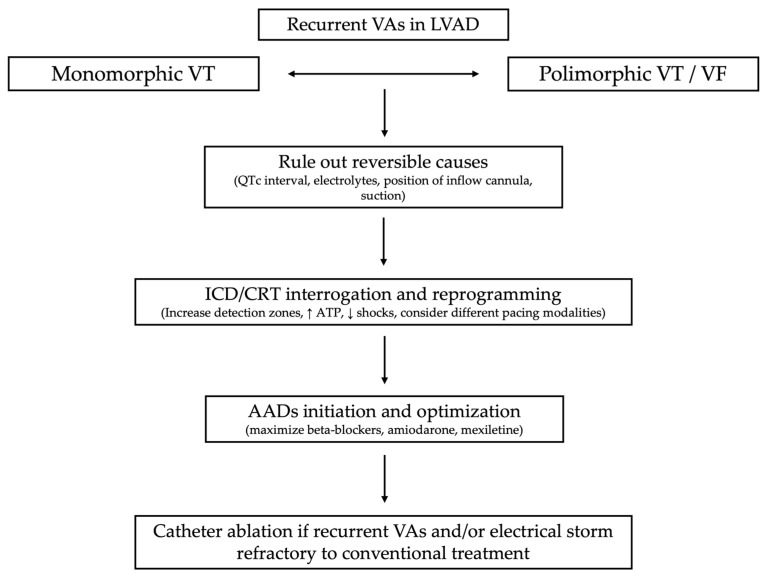
A workflow algorithm showing arrhythmia management in LVAD patients.

Therapeutic options such as catheter ablation may be considered as part of a comprehensive approach. The optimal timing of treatment and whether catheter ablation should be performed before or after LVAD implantation remains unclear. Several factors should be evaluated in the decision-making process within a multidisciplinary team setting, including HF specialists, electrophysiologists, cardiothoracic surgeons, nurses, and an LVAD coordinator [2]. The most widely accepted indications for catheter ablation in LVAD patients are incessant VT, electrical storm, recurrent ICD interventions, or progressive RV failure due to VAs. A workflow diagram is shown in Figure 6.

### 7.1. Ablation of VAs Before LVAD Implantation

As previously discussed, a significant history of VAs before LVAD implantation strongly predicts post-implant VAs [9,25,26,27,28,29]. Therefore, performing prophylactic ablation in these patients may reduce the occurrence of VAs after LVAD surgery, which complicates the early postoperative period [3]. Catheter ablation may help stabilize the patient’s hemodynamics and, as a result, could potentially delay or prevent the need for LVAD implantation. In addition, pre-LVAD ablation may be advisable in all cases where the hypothesized VA substrate, such as the epicardium, will not be readily accessible after an LVAD implant.

However, given the advanced stage of the underlying disease, completely removing the substrate responsible for the VAs may be difficult. Many authors think that prophylactic VA ablation before LVAD implantation could be considered unnecessary treatment. Patients suitable for LVAD implantation have end-stage HF with very low left ventricular ejection fraction. They are often frail and at risk of sudden peri-procedural hemodynamic decline, needing intravenous vasopressors and inotropic agents to support cardiac output during the procedure or even temporary mechanical support. Therefore, if pre-LVAD catheter ablation is considered, careful and personalized risk assessment before the procedure is essential to reduce the risk of peri-procedural complications. 

### 7.2. Ablation of VAs During LVAD Implantation

The main advantage of performing VA ablation during LVAD implantation is the ability to combine epicardial and endocardial ablation, which generally yields better results than standard transcatheter endocardial ablation [47,48,49]. During LVAD implantation, the epicardium is exposed and easily accessible for peri-implant mapping and transcatheter or surgical ablation of VAs. After LVAD implantation, however, epicardial access for catheter ablation becomes limited due to post-surgical adhesions and the position of the LVAD pump itself. Consequently, peri-implant transcatheter or surgical ablation has been suggested for patients with recurrent VAs and a suspected epicardial substrate. Intraoperative ablation can be guided by preoperative imaging, image integration, electrophysiological study, and intraoperative mapping [50,51,52,53,54,55]. Moss et al. described the feasibility of high-density electro-anatomical mapping in an open-chest model to characterize epicardial substrate during LVAD implantation. Although some limitations were inherent to impedance-based mapping technology and the small sample size, this study provided preliminary data indicating that epicardial electro-anatomic mapping may be helpful for risk stratification in patients undergoing LVAD implantation [53]. This supports the need for a prospective investigation into the empiric epicardial ablation of potentially arrhythmogenic substrates at the time of LVAD implantation. More information regarding the timing of ablation will become available after the current PIVATAL trial concludes. This prospective randomized trial includes patients with a history of previous VA. It will compare peri-implantation VA ablation with conventional medical therapy, with the primary endpoint being overall VA episodes [56]. 

### 7.3. Ablation of VAs After LVAD Implantation

In patients with recurrent VA episodes after LVAD implantation, a removable cause should be identified. VAs may be linked to the inlet cannula irritation or LV wall suction, which is even more probable when VAs are associated with hemolysis or are respiratory-dependent or linked with cough [21]. LVAD interrogation and transthoracic or transesophageal echocardiography assist in diagnosing these phenomena. Reprogramming the LVAD and/or optimizing LV volume may be enough to stop VAs. The timing of VAs should also be considered before proceeding with catheter ablation. Early post-surgical VAs are pretty common and may also be triggered by inotropic or vasopressor drug support. Early VAs are generally self-limiting, and treatment with AADs is usually sufficient. A more conservative approach can be adopted in patients whose LVAD is a BT. Conversely, in patients undergoing LVAD implantation as a DT, the risk of VA recurrence is higher, and catheter ablation might be a reasonable option. 

### 7.4. Pre-Procedure Planning and Technical Considerations

Several additional considerations need to be considered when planning a catheter ablation in patients with an LVAD. Pre-procedural imaging is valuable for defining the inflow cannula projection into the LV and can provide essential information regarding the arrhythmic substrate. Cardiac magnetic resonance imaging cannot be performed in LVAD patients. Computed tomography (CT) and echocardiography are the preferred imaging techniques in such patients. Additionally, the pre-procedural CT scan can be segmented and analyzed using specialized software (inHEART Model Shaper v1.1.1, inHEART 33600 Pessac France; or ADAS 3DLV, https://www.adas3d.com/about-us/, access date: 14 September 2025), and the resulting 3D model, which contains information about the underlying substrate, can be merged with the 3D-electroanatomic map (3D-EAM) [57].

Maintaining a proper level of anticoagulation without interruption during ablation in LVAD patients is essential to reducing the substantial risk of thromboembolism. In the case series reported in the literature, INR was kept between 2.5 and 3.5, or when UFH was preferred, an ACT >300 s was maintained [21].

Ultrasound guidance for vascular access has become standard practice to reduce complications, but it is even more advantageous in LVAD recipients due to diminished or absent peripheral pulses. When accessing the left ventricle (LV), transseptal puncture should be considered the primary choice. This approach avoids the outflow cannula and output graft in the aorta. It is preferable because retrograde access to the LV cavity can be difficult due to the reduced opening of the aortic valve. Retrograde aortic access may be selected if transseptal access cannot effectively reach target areas such as the basal septum and inferior LV segments [58]. 

**Figure 7:**
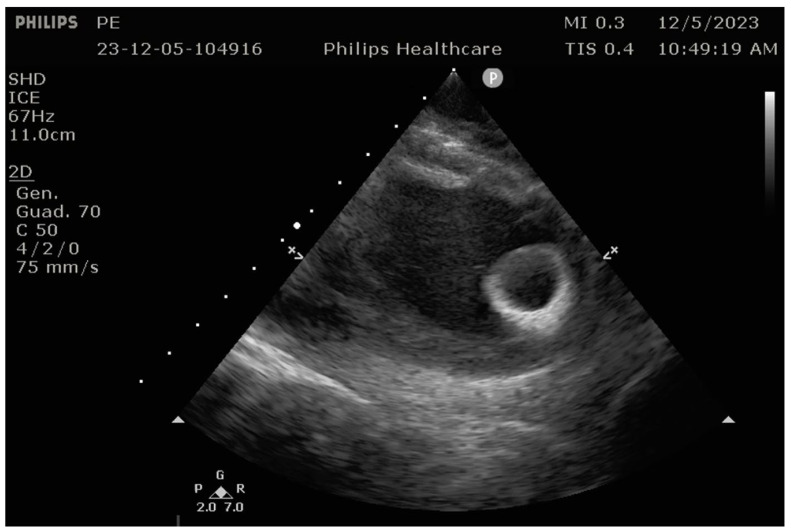
Intracardiac echocardiography during ventricular tachycardia ablation in a patient with LVAD. The apical inflow cannula is well evident.

Although reprogramming the LVAD is not usually necessary before and after the procedure, in certain cases, temporary reprogramming can be beneficial [29]. For instance, due to the suction effect of the LVAD, the LA volume might decrease, requiring adjustments to the LVAD settings to promote atrial distension before transseptal puncture to access the LV [57]. The reduction in LV volume caused by LVAD activity must be considered during catheter ablation, as it can affect catheter maneuverability, particularly around the apical inflow cannula [59,60]. Catheter entrapment by the inflow cannula is a significant concern during catheter ablation in LVAD recipients, though the incidence of this complication remains low. Beyond fluoroscopy, ICE-guided ablation plays an important role in preventing catheter entrapment [23]. In complex procedures, ICE has been shown to enhance safety by providing real-time imaging, improving catheter-tissue contact, aiding in substrate and target identification, and reducing fluoroscopy times [11,23] (Figure 7). 

### 7.5. Intraprocedural Considerations

Several essential aspects must be considered during the ablation of VAs in LVAD recipients. The high-speed revolutions of the central electromagnetically driven centrifugal rotor may create EMI with the magnet-based 3D-EAM systems. Similarly, EMI has also been reported in patients with LVAD and apical ICD lead. EMI can be significantly reduced or avoided if the ICD lead is implanted into a septal area [61]. During catheter ablation, EMI typically occurs when the mapping or ablation catheter is within 4 cm of the apical inflow cannula, which can reduce the quality of EGM recordings, catheter location accuracy, point acquisition, vector orientation, and contact force sensing readings [62]. Reducing the flow rate from 1.9 to 2.5 L/min to 1.4 to 2.0 L/min has effectively eliminated severe EMI [22]. LVAD provides hemodynamic stability during sustained VT, even under general anesthesia. However, some groups prefer to perform the procedure under conscious sedation to maximize the likelihood of VT inducibility [63]. During sustained VT, activation mapping using multi-electrode mapping catheters and entrainment mapping are effective strategies for identifying the VT substrate. Anderson et al. reported the adoption of activation mapping and entrainment mapping in 60% of cases, substrate mapping in 20% of cases, and a combination of both approaches in the remaining 20%, which differs from non-LVAD patients, in whom the VAs ablation is predominantly guided by substrate mapping [22]. Patients with LVAD commonly have a large area of endocardial scar and late potentials. Therefore, scar homogenization and late potential abolition can be challenging. Functional substrate mapping, such as isochronous late activation mapping during paced or sinus rhythm, or decremental evoked potential mapping, may help localize regions within the scar most likely to be involved in VT circuits, eliminating the need for more extensive substrate-based ablation.

Although the extent of endocardial scar has recently been associated with VT recurrence [64], it is still unclear whether ablation aimed at the complete elimination of all inducible VTs is always achievable and would not lead to an excessive increase in procedure-related complications. As such, the primary goal of the ablation should be the elimination of the clinical VT [2]. In the recent consensus statement, non-inducibility of the clinical VT was achieved in 78% of cases [2].

### 7.6. Procedure-Related Complications and Recurrences

Major procedural complications are uncommon, likely because experienced operators perform these high-risk cases. In centers with experience in VT ablation, the incidence and type of procedural complications associated with VT ablation in LVAD recipients are comparable to or slightly higher than those in non-LVAD patients [65]. In the meta-analysis by Anderson et al., the overall incidence of complications was 9.4%, with major complications occurring in 5.5% of cases. Most complications were comparable to those identified in non-LVAD VT ablation and associated with vascular access sites or thromboembolic events. LVAD-related specific complications are catheter entrapment, pump thrombosis, and post-procedure persistent atrial shunting. Although to date, no cases of catheter entrapment in the inflow cannula have been reported, extreme caution is needed when mapping close to the inflow apical cannula of the LVAD. Catheter ablation has been reported to be an independent predictor of pump thrombosis [66]. The risk of pump thrombosis and thromboembolic increases immediately after VT ablation and may remain increased for several weeks. Specific caution is advised when mapping and ablating around the inflow cannula, as this appears to be associated with the highest incidence of pump thrombosis [67].

Finally, rare cases of persistent iatrogenic (after transseptal) atrial septal defect with right-to-left shunting with hypoxemia have been reported [68,69]. Transesophageal echocardiography helps identify these cases, and percutaneous atrial septal occlusion may be required to prevent consequences. Complications reported in the literature are listed in Table 2.

Anderson et al. reported a VT recurrence in 43.6% of patients, but there was a significant reduction in ICD therapies or shocks [22]. The recently published CHANNELED registry reported a VT recurrence in 55% of patients with no difference between nonischemic and ischemic cardiomyopathy patients (46% vs. 61%; log rank P = 0.19) [64]. Primary reasons for the failure of endocardial ablation include the presence of epicardial or deep intramural VA substrates. Alternative strategies to target epicardial substrate include noninvasive cardiac radioablation or surgical ablation. Radioablation is currently considered a bailout strategy in refractory cases that have previously failed multiple ablation attempts, in substrates considered unreachable, or in patients deemed poor catheter ablation candidates [63].

## 8. Conclusions

VAs are common among patients with LVAD and significantly contribute to increased morbidity and mortality in this population. A current evidence-based workflow algorithm for VAs management in LVAD patients is shown in Figure 7. A history of VAs prior to LVAD implantation is a strong predictor of both early and late onset of VAs after the procedure. The primary cause of VAs is scar-related macro-reentry within existing scar tissue, while VAs associated with inflow cannulas account for only a small percentage of cases. The first-line treatments for VAs in LVAD recipients are ICD and AAD therapy. Optimizing ICD programming to reduce the frequency of shocks is crucial for the management of these patients. In case of failure, catheter ablation is a safe and effective treatment in this high-risk population. Ablation is effective in terminating acute ES and, overall, results in a reduction in defibrillator shocks. Acute procedural success, complications, and recurrences are comparable to a non-LVAD population. Epicardial mapping and ablation after LVAD implantation are challenging due to pericardial adhesions, and alternative strategies to target the epicardial substrate should be considered. Surgical epicardial and endocardial ablation during LVAD implantation can help reduce the VT burden after LVAD implantation.

## 9. Future Directions

Despite significant advancements in mechanical circulatory support over the past few decades, there are still considerable challenges that affect clinical outcomes and quality of life. Current studies aim to provide more insight into optimal management of arrhythmias, particularly regarding the optimal timing of catheter ablation. Non-invasive mapping software can visualize VT in real-time for patients with LVAD, offering valuable information for personalized treatment aiding in procedural planning alongside pre-procedural imaging. Lastly, future directions will focus on optimizing ICD programming, which includes pacing modes and shock thresholds, to ensure proper hemodynamics and device performance.

## Figures and Tables

**Table 1:** Recommended ECG filter settings for currently available continuous-flow left ventricular assist devices (LVADs).

LVAD Model	Typical Pump Speed(rpm)	Main EMI Frequency (Hz)	Recommended Low-Pass Filter	Band-Stop Filter Option	Notes/Tips
**HeartMate 3 (Abbott)**	5000–6000 rpm	~83–100 Hz	Set low-pass ≈40 Hz; preserves QRS morphology but may attenuate ST segments	Band-stop at pump-specific peak (~83–100 Hz)	Multiple peaks may occur due to the pump’s artificial pulsatility; more than one notch filter may be required
**HeartMate II (Abbott)**	8800–10,000 rpm	~147–167 Hz	Low-pass 40–60 Hz usually sufficient; artefact occurs at high frequencies	Notch filter rarely needed unless peaks overlap power-line frequency	High-frequency artefacts; lowering the low-pass filter to ≈40 Hz almost always improves ECG clarity
**HeartWare (Medtronic)**	2400–3200 rpm	~40–53 Hz	Set low-pass ≤40 Hz to suppress primary artefact	Optional: notch filter around 40–50 Hz	Artefacts usually occur at lower frequencies and are easily managed by lowering the low-pass filter

**Table 2:** Complications of ventricular tachycardia ablation reported in the literature.

Complication	Incidence % (n)	Reference
Groin hematoma	3.6–4.4% (4/110)	Anderson et al. [22]
Vascular access surgically treated	1.8% (2/110)	Anderson et al. [22]
Cerebrovascular accidents	1.8% (2/110)	Anderson et al. [22]
Cardiogenic shock	0.9% (1/110)	Anderson et al. [22]
Pump thrombosis	Rare to 11%	Anderson et al. [22] Grinstein et al. [66]
Persistent ASD with right-to-left shunt	Rare	Wang et al. [68] Tamura et al. [69]
Catheter entrapment in LVAD cannula	Not yet reported for LVAD	

Legend. ASD = atrial septal defect.

## Data Availability

The data presented in this study are available upon reasonable request.

## Abbreviations

The following abbreviations are used in this manuscript:

VAs	Ventricular Arrhythmias
LVADs	Left Ventricular Assist Devices
ICDs	Implantable Cardioverter Defibrillators
DT	Destination Therapy
AADs	Antiarrhythmic Drugs
BT	Bridge to cardiac Transplantation
HF	Heart Failure
VF	Ventricular Fibrillation
CRT-Ds	Cardiac Resynchronization Therapy with Defibrillators
CIEDs	Cardiac Implantable Electronic Devices
CT	Computed tomography
ICE	Intra-Cardiac Echography
3D-EAM	Three-Dimensional Electroanatomic Map
EMI	Electro Magnetic Interference
